# MOMENTARY UNCONSTRUCTIVE REPETITIVE THINKING, WORKING MEMORY, AND AGE

**DOI:** 10.1093/geroni/igae098.2707

**Published:** 2024-12-31

**Authors:** Julie Kircher, Eric Cerino, Jacqueline Mogle, Susan Charles

**Affiliations:** The Pennsylvania State University, State College, Pennsylvania, United States; Northern Arizona University, Flagstaff, Arizona, United States; Clemson University, Clemson, South Carolina, United States; University of California Irvine, Irvine, California, United States

## Abstract

Both trait and daily negative affect are often associated with lower performance on working memory tasks, perhaps because negative affect is often related to unconstructive repetitive thinking (URT) – negative ruminative thoughts about oneself or the environment. URT is posited to deplete attentional resources, resulting in downstream effects on cognition. Studies examining the effects of URT on cognitive performance often use laboratory-based study designs. This study aimed to investigate how reports of URT throughout the day are linked to scores on momentary cognitive tasks of verbal working memory. We hypothesized that reports of greater engagement in URT will be related to worse working memory, and that this association was related to age. Data from the Effects of Stress on Cognitive Aging, Physiology, and Emotion (ESCAPE) study included 263 community residents ranging from 25 to 65 years old (Mage = 46.5) completing five assessments throughout the day across 14 days. Contrary to our hypothesis, we found that greater URT was related to improved verbal working memory (γ =.005, SE =.001, p <.001). Higher momentary negative affect was related to poorer working memory, acting as a suppressor on the benefits of URT on working memory performance. This relationship between URT and verbal working memory remained significant across ages, but the relationship between levels of URT and working memory was strongest among younger adults.

